# CANAGLIFLOZIN INCREASES INTESTINAL ADENOMA BURDEN IN FEMALE APCMIN/+ MICE

**DOI:** 10.1093/geroni/igad104.0066

**Published:** 2023-12-21

**Authors:** Justin Korfhage, David Lombard

**Affiliations:** Yale School of Medicine, New Haven, Connecticut, United States; University of Michigan, Ann Arbor, Michigan, United States

## Abstract

DOI: 10.1093/gerona/glab254

